# Synergistic Synbiotic-Containing *Lactiplantibacillus plantarum* and Fructo-Oligosaccharide Alleviate the Allergenicity of Mice Induced by Soy Protein

**DOI:** 10.3390/foods14010109

**Published:** 2025-01-02

**Authors:** Jing Bai, Qian Zeng, Wen Den, Liheng Huang, Zhihua Wu, Xin Li, Ping Tong, Hongbing Chen, Anshu Yang

**Affiliations:** 1 State Key Laboratory of Food Science and Resources, Nanchang University, Nanjing Dong Lu 235, Nanchang 330047, China; jiebenbai@163.com (J.B.); zq2831164447@163.com (Q.Z.); 13340115395@163.com (W.D.); hlhncu@163.com (L.H.); wuzhihua@ncu.edu.cn (Z.W.); zhizilixin@ncu.edu.cn (X.L.); tongping@ncu.edu.cn (P.T.); chenhongbing@ncu.edu.cn (H.C.); 2 School of Food Science and Technology, Nanchang University, Nanchang 330047, China; 3 Sino-German Joint Research Institute, Nanchang University, Nanjing Dong Lu 235, Nanchang 330047, China

**Keywords:** fructo-oligosaccharides, *Lactiplantibacillus plantarum*, dietary intervention, mouse model, food allergy

## Abstract

Prebiotics and probiotics have key roles in the intervention and treatment of food allergies. This study assesses the effect of *Lactiplantibacillus plantarum* synergistic fructo-oligosaccharide (Lp–FOS) intervention using an allergic mouse model induced by soy protein. The results showed that Lp synergistic FOS significantly decreased clinical allergy scores, inhibited specific antibodies (IgE, IgG, and IgG1), IL-4, IL-6, and IL-17A levels, and increased IFN-γ and IL-10 levels. Meanwhile, flow cytometry showed that Lp–FOS intervention inhibited the percentage of dendritic cell (DC) subsets in splenocytes and increased the Th1/Th2 and Treg/Th17 ratios. Furthermore, Lp–FOS intervention upregulated the mRNA levels of T-bet and Foxp3 and downregulated the mRNA levels of GATA3. Finally, non-targeted metabolomic analysis showed that Lp–FOS improved serum metabolic disorders caused by food allergies through regulating glycine, serine, and threonine metabolism, butanoate metabolism, glyoxylate and dicarboxylate metabolism, the biosynthesis of cofactors, and glycerophospholipid metabolism. These data showed that the combination formulation Lp–FOS could be a promising adjuvant treatment for food allergies.

## 1. Introduction

A food allergy is an adverse immune reaction that occurs when the body ingests certain specific foods [1,2]. Its clinical symptoms include oral hypersensitivity syndrome, urticaria, intestinal disorders, asthma, and allergic rhinitis, which can lead to anaphylactic shock or even death in extreme situations [3]. Recently, food allergies have become an inescapable food safety issue worldwide [4,5,6]. Soybean is one of the main food allergens identified by the WHO/FAO and it causes an immunoglobulin (Ig) E-mediated type I hypersensitivity reaction [7,8]. The main reason is that soybeans contain allergy proteins such as glycinin and β-conglycinin, the content of which can reach more than 70% of the total soybean protein [9,10]. Meanwhile, soybean, as an important raw material of plant-based food, will also greatly increase the risk of food allergies caused by the intake of soy products.

Oligosaccharides are a type of low-molecular-weight carbohydrates typically composed of approximately 10 monosaccharides, which are linked by glycosidic bonds to form straight or branched chains of polymers. Studies have shown that some functional oligosaccharides have prominent roles in promoting the proliferation of probiotics, enhancing immunity, and preventing intestinal diseases [11,12,13]. It was shown that fructo-oligosaccharide (FOS) alleviated OVA-induced intestinal inflammation and allergic symptoms by inhibiting the release of Th2 cytokines, activating initial CD4 T cells, and improving the balance of T regulatory cells (Tregs) and T helper (Th) 17 cells in mice [14]. An allergic mouse model induced by whey demonstrated that dietary intervention with short-chain galactose, long-chain fructose, and acidic oligosaccharides diminished acute allergic skin reactions and inhibited the partial depletion of CD25+ T cells [15].

*Lactobacillus* is a Gram-positive bacterium that ferments carbohydrates (mainly glucose) to produce a large amount of organic acid. As an important member of the gut microbiota, *Lactobacillus* could modulate the intestinal environment and improve intestinal health [16,17]. There are many reports about *Lactobacillus* alleviating allergic reactions by influencing the host’s immune system [2]. A study showed that *Lactobacillus reuteri* enhanced the intestinal immune tolerance of food allergies in mice, such as by suppressing the Th2 immune response and upregulating the percentage of tolerogenic dendritic cells (DCs) and Tregs in the intestine [18]. Oral administration of VSL#3 significantly reduced shrimp myosin-induced hypersensitivity in mice by effectively strengthening the Th1 reaction and reducing IgE levels [19]. Studies have shown that gavage of *Bifidobacteria* significantly inhibited the Th2 response and increased the percentage of Tregs in OVA-induced allergic mice. In addition, transient translocation of *Bifidobacterium* was observed in mice [20].

Non-targeted metabolomic profiling has been used to dissect the pathogenesis of complex diseases, and it was successfully used to characterize allergic asthma, and the metabolic biomarkers related to disease severity and type were identified [21,22]. Recently, many studies have used multi-omics strategies to study food allergies [23,24].

Currently, most research has concentrated on the health benefits of a single prebiotic or probiotic. It was believed that prebiotics could induce probiotics to colonize the gut and enhance immune functions, thus alleviating allergic symptoms and preventing the risk of allergies developing [25,26,27]. However, synbiotics composed of specific probiotics and selected prebiotics are generally more effective than probiotics or prebiotics alone from the perspective of gut health and function [28,29]. The modulation mechanisms of probiotics, prebiotics, and synbiotics are extremely complicated, involving the interaction of signaling pathways, immune cells, and their receptors. Furthermore, these interactions could stimulate the activation and maturation of DCs and induce native T cell differentiation, which mainly upregulates the Th1/Th2 ratio and Tregs [30]. Nevertheless, the existing findings are inconsistent and the mechanism underlying food allergies remains unclear. In addition, there are few basic studies on soybean allergy intervention compared with other common allergens. Here, a type I food allergy mouse model sensitized with soy protein was established to evaluate the intervention of *Lactiplantibacillus plantarum* (Lp) in synergy with FOS. This study will fill the gap in soybean allergy research on synergistic intervention with combined probiotics with oligosaccharides and provide novel perspectives for the clinical intervention and treatment of soybean allergies using synbiotics in the future.

## 2. Materials and Methods

### 2.1. Materials

Soybean ‘Dongnong 48’ was contributed by Northeast Agricultural University, China. *Lactiplantibacillus plantarum* CICC 20988 was acquired from the Industrial Microbiology Collection Management Centre, China. Cholera toxin (CT, 95%) was purchased from Sigma-Aldrich (St Louis, MO, USA). The mouse splenocyte enzyme-linked immunosorbent assay (ELISA) kits were obtained from Invitrogen (Thermo Fisher Scientific, Waltham, MA, USA). Goat anti-mouse specific IgG, IgG1, and IgE antibodies were all acquired from eBioscience (Thermo Fisher Scientific, Waltham, MA, USA). Flow cytometry antibodies were acquired from BioLegend (San Diego, CA, USA).

BALB/c mice (Female, SPF) were purchased from GemPharmatech Co., Ltd. (Nanjing, China) and raised in standard circumstances (24 ± 2 °C, 50 ± 10% humidity). All mice drank and ate (AIN93G meal devoid of soybean) freely. This study was conducted following the guidelines authorized by the Animal Ethical Committee of Laboratory animal Science and Technology center, Nanchang University, China (NCULAE-20240904003).

### 2.2. Sample Preparation

Soybean protein was prepared according to the method of Yang et al. [31].

### 2.3. Protocol of Experiments

The animal model was designed based on previous studies with some modifications [32]. Second-generation BALB/c female mice (5–6 weeks) were used in this study. The five experimental groups (n = 9/group) consisted of control (PBS group), soy protein (soy protein-sensitized model group), Lp (Lp intervention group), FOS (FOS intervention group), and Lp–FOS (Lp + FOS intervention group) groups. The mice in the control group were administered PBS (only PBS treatment). On days 0, 7, 14, and 21, the mice in the soy protein group and other intervention groups were administered 5 mg soy protein and 10 μg CT. Meanwhile, Lp, FOS and Lp–FOS groups were administered Lp (10^8^ CFU/day), FOS (300 mg/kg/day), and Lp–FOS [Lp (10^8^ CFU/day + FOS (300 mg/kg/day)] every day for 20 days, respectively. On day 28, soybean protein (20 mg) was used to challenge the mice. At 30–60 min after the stimulation of intragastric administration, clinical allergic symptoms were observed and the body temperature was measured. On day 29, serum was collected to detect the indicators of food allergy. After all the mice were euthanized, the spleens and intestinal tissues were collected for a comprehensive allergenicity assessment.

### 2.4. Clinical Score, Body Weight, and Temperature

Clinical allergy scores were recorded 30 min after the mice were challenged (Table 1) [33]. Then, changes in body temperature and weight were recorded.

### 2.5. Detection of Specific Antibodies

The blood of mice was collected and stored for 2 h. After centrifugation (8000× *g*, 10 min), the serum of mice was collected.

The specific antibody concentration in the mouse serum was measured using an indirect ELISA. A soy protein solution was used to coat the 96-well microplate, which was stored at 4 °C overnight. Next day, after discarding the uncoated solution, a skimmed milk powder solution (5%) was added to the wells and incubated (37 °C, 1 h). Serum (diluted ratios were 1:20, 1:20, and 1:200) was added to detect IgE, IgG, and IgG1 levels, respectively. After washing and discarding the blocking solution, the secondary antibodies (100 μL/well, IgE, IgG, and IgG1, diluted 1:10000) and HRP-streptomycin-labeled avidin (100 μL/well, diluted ratio was 1:60) were added. Furthermore, 3,3′,5,5′-tetrame-thylbenzidine was added and incubated (37 °C, 15 min) in the dark. Finally, the reaction was terminated by adding 50 μL 2 M H_2_SO_4_, and a Bio-Rad 1860 microplate reader was used to detect the absorbance at 450 nm.

### 2.6. Detection of Splenocyte Cytokines

Mouse spleens were aseptically isolated and pulverized with RPMI 1640 medium on a 70 µm cell sieve. After lysing the red cells, splenocytes were resuspended and adjusted to 5 × 10^6^ mL^−1^. Then, soy protein was added to stimulate splenocytes in the incubators (37 °C, 5% CO_2_) for 72 h. Finally, commercial ELISA kits were used to detect the released cytokines of splenocytes (IL-4, IL-6, IL-10, IL-17A, and IFN-γ).

### 2.7. Detection of Immune Cells

According to Section 2.6, the splenocytes (5 × 10^6^ mL^−1^) were obtained. Zombie Green™ Fixable Viability and Fc Block™ were added and incubated (4 °C, 30 min). Then, the supernatant was discarded after centrifugation (500× *g*, 5 min).

(1) For DCs, the cells were incubated with mixed mouse antibodies (CD86-PE, CD40-PE-Cy7, CD11c-APC, CD103-BV605, and MHCII-BV421) in the dark for 30 min.

(2) For T lymphocyte subsets, the cells were incubated with mouse CD4-PerCP and mouse CD25-PE-Cy7 antibodies in the dark for 30 min. After centrifugation (500× *g*, 5 min), the cells were mixed with fixative and rupture agents. Then, the cells were incubated with mixed mouse antibodies (T-bet-BV421, GATA3-PE, Foxp3-AF647, and RORγt-BV650) in the dark for 30 min.

Finally, the TF Perm/wash buffer was added to wash the cells. After centrifugation, the cells were resuspended with PBS, and the DCs, Th1 cells, Th2 cells, Tregs, and Th17 cells were detected by flow cytometry.

### 2.8. RT-qPCR

For mRNA isolation, the jejunum of the mice was pulverized in TRIzol RNA lysis buffer (containing three zirconia grinding beads). Chloroform was added to the jejunal tissue fluid. Isopropanol was added to the supernatant after centrifugation (10,000× *g*, 15 min, 4 °C). The precipitate was washed with 75% ethanol after centrifugation. Finally, the cleaned RNA precipitate was dissolved in an appropriate amount of sterile, enzyme-free water. RNA content was measured using trace amounts of nucleic acid protein detector. The purity of RNA was assessed by the 260/280 nm and 260/230 nm ratios. Furthermore, the isolated RNA was reverse-transcribed into cDNA.

The primer sequences were synthesized by Shanghai Biotechnology Bioengineering Technology Service Co., Ltd. (Table S1).

For qPCR, isolated RNA, which was added to the primers, and ChamQ Universal SYBR qPCR Master Mix were amplified using the Quant Studio™ Real-Time PCR Detection System. The mRNA expression of each gene was normalized by GAPDH expression as a reference, and the mRNA expression of each mouse was represented as a fold change of the average in the control group.

### 2.9. Non-Targeted Metabonomic Profiling of Serum

Serum metabolites were extracted using a methanol solution. The mixture underwent ultrasound at 5 °C (40 kHz, 30 min). After centrifugation (13000× *g*, 15 min) at 4 °C, the supernatant was analyzed by LC-MS/MS.

Chromatographic conditions: Column is HSS T3 (100 mm × 2.1 mm i.d., 1.8 μm), injection volume is 2 μL, column temperature is 40 °C, and the flow rate is 0.4 mL/min. The mobile phase A is formic acid and acetonitrile (95:5, *v*/*v*). The mobile phase B is formic acid in a solution of acetonitrile, isopropanol, and water (47.5:47.5:5, *v*/*v*).

MS conditions: Data collection was performed in the Data-Dependent Acquisition (ADD) mode. Detection was conducted within the 70–1050 *m*/*z* mass range. Other specific detected conditions were slightly modified according to previous studies [34,35].

Progenesis QI (Waters Corporation, Milford, CT, USA) software was used to preprocess raw LC/MS data, and a three-dimensional data matrix was transformed into the CSV format. After the metabolites were searched and identified in the main databases of KEGG (https://www.kegg.jp/kegg/compound/, accessed on 30 August 2023) and Majorbio Database, the data were analyzed by transferring it onto the Majorbio cloud platform (https://cloud.majorbio.com).

### 2.10. Statistical Analysis

The results were presented as mean ± SEM, and the data were processed by SPSS 22.0. One-way analysis of variance was performed. *p* < 0.05 indicates significant differences.

## 3. Results

### 3.1. Mouse Allergy Score, Body Temperature, and Weight

**Figure 1:**
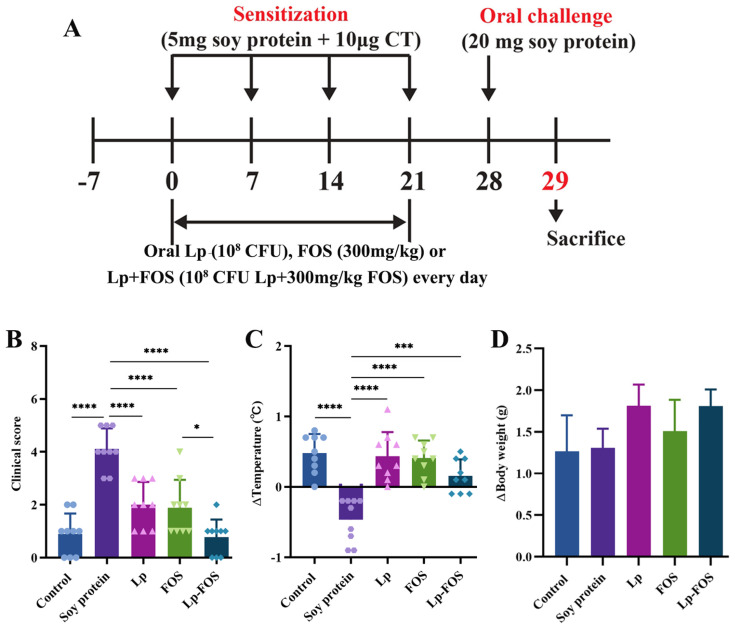
Intervention effect of Lp and FOS on mice allergic to soybeans. (**A**) Schematic diagram of mice with soy allergies and their preventive model. (**B**) Clinical score of acute hypersensitivity. (**C**) Body temperature. (**D**) Weight gain. * *p* < 0.05, *** *p* < 0.001, and **** *p* < 0.0001.

The BALB/c mouse has been commonly used to investigate hypersensitive diseases due to its near-crossing high IgE response. The synergistic intervention effect of Lp and FOS on allergic reactions was assessed using a soy protein-induced mouse model, including acute allergy symptom scores, changes in body temperature, and weight. In terms of allergy scores, severe allergic symptoms were observed in the soy protein group (Figure 1B), with significant differences from other groups (*p* < 0.0001). Furthermore, the rectal temperatures of mice were measured after challenge by soy protein (Figure 1C). It revealed that the temperature of mice was significantly decreased (*p* < 0.0001) after stimulation by the allergic protein (soy protein). In terms of the weight of the mice, no significant differences were observed in all groups, while most mice in the soy protein group tended to lose weight compared to the other groups (Figure 1D). These findings affirmed that the allergic mouse model induced by soy protein was successfully established.

Furthermore, the administration of Lp and FOS (with no significant difference) significantly reduced clinical scores (*p* < 0.0001). It is essential to highlight that these significant differences were observed in the FOS and Lp–FOS groups (*p* < 0.05). The body temperatures of mice in the Lp, FOS, and Lp–FOS groups were significantly higher compared with the soy protein group, and they had no significant differences with the control group. Additionally, the body temperatures of mice in the Lp, FOS, and Lp–FOS groups had no discernible disparity (*p* > 0.05). In summary, these results suggested that Lp synergistic FOS could more effectively alleviate allergic symptoms in mice.

### 3.2. Serum-Specific Antibody Levels

**Figure 2:**
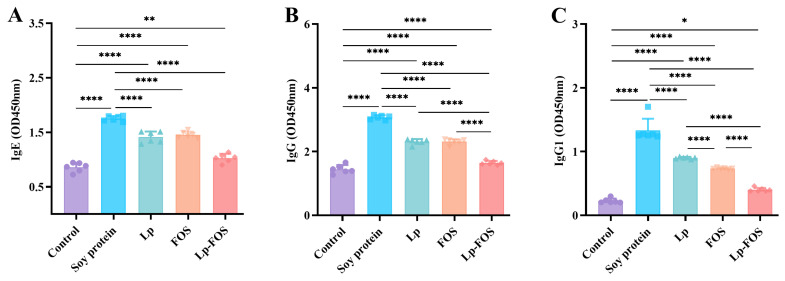
Serum-specific antibody levels in mice. (**A**) IgE. (**B**) IgG. (**C**) IgG1. * *p* < 0.05, ** *p* < 0.01, and **** *p* < 0.0001.

Most food allergies are classified as type I hypersensitive reactions, which mainly include an obvious upregulation of the serum-specific antibodies IgE, IgG, and IgG1. Soy protein-specific antibodies were further detected in the serum of mice (Figure 2). Significantly increased serum-specific IgE, IgG, and IgG1 levels were observed in the soy protein group (*p* < 0.0001). Figure 2A showed that the levels of IgE were lower in the Lp, FOS, and Lp–FOS groups than in the soy protein group and higher than in the control group. No significant differences were observed in the Lp, FOS, and Lp–FOS groups, but the Lp–FOS group had the lowest level of IgE. IgG levels in the serum of mice were significantly reduced in the Lp, FOS, and Lp–FOS groups, while they were also higher than in the control group (Figure 2B). IgG1 levels were similar to IgG levels in mice serum. However, the level of IgG1 in the Lp–FOS group exhibited a significant reduction in comparison with the Lp and FOS groups (*p* < 0.0001, Figure 2C). Interestingly, IgG and IgG1 levels in the serum of the Lp–FOS group were all markedly decreased. Thus, these results revealed that Lp–FOS could exert synergistic effects to downregulate the levels of specific antibodies caused by a soybean allergy.

### 3.3. Cytokines

**Figure 3:**
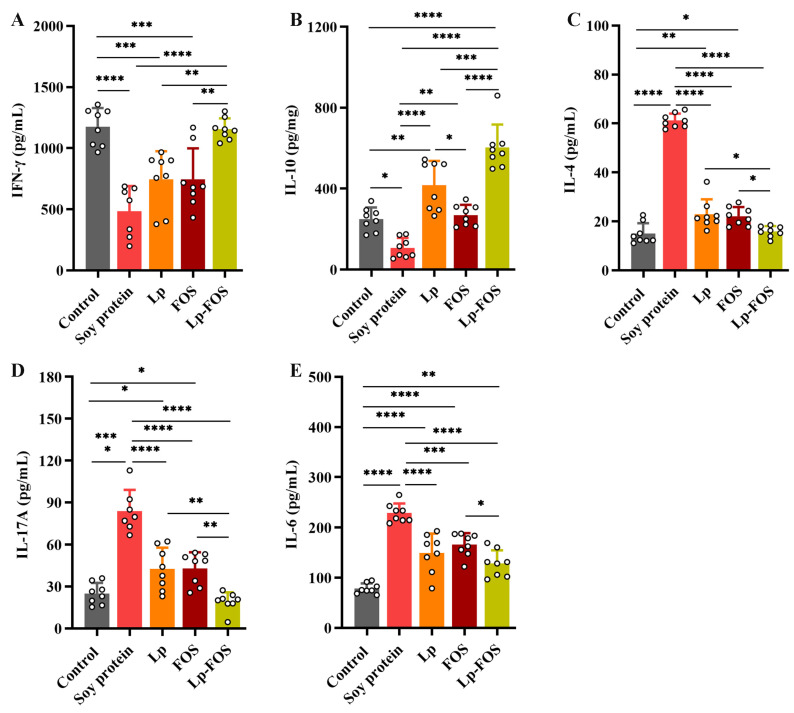
The levels of cytokines in mice. (**A**) IFN-γ. (**B**) IL-10. (**C**) IL-4. (**D**). IL-17A. (**E**) IL-6. * *p* < 0.05, ** *p* < 0.01, *** *p* < 0.001, and **** *p* < 0.0001.

The results of in vitro stimulation of cytokine levels in the mouse splenocytes are shown in Figure 3. IFN-γ and IL-10 concentrations in the splenocyte supernatant of the soy protein group were significantly decreased (*p* < 0.0001 and *p* < 0.05, respectively, Figure 3A,B). In comparison with the soy protein group, the FN-γ concentration of the splenocyte supernatant was increased in the Lp, FOS, and Lp–FOS groups. However, only the Lp–FOS group displayed a significant elevation in comparison with the soy protein group (*p* < 0.0001). Meanwhile, IFN-γ concentrations in mice in the Lp–FOS group were markedly elevated compared with the Lp and FOS groups (*p* < 0.01, Figure 3A). In the Lp, FOS, and Lp–FOS groups, the IL-10 concentration of the splenocyte supernatant was also upregulated compared with the soy protein group, and the IL-10 concentration was highest in the Lp–FOS group (Figure 3B). Furthermore, the IL-4, IL-6, and IL-17A concentrations of the splenocyte supernatant in the soy protein group were significantly upregulated (Figure 3C–E, *p* < 0.0001). The concentration of IL-4 in the supernatant of mice from the Lp, FOS, and Lp–FOS groups was markedly decreased compared with the soy protein group, while no significant differences in IL-4 levels were observed in the Lp and FOS groups (Figure 3C). IL-6 and IL-17A concentrations in the splenocyte supernatant were similar to IL-4, while the concentrations of IL-4 and IL-17A in the splenocyte supernatant had no significant differences between the Lp–FOS and control groups (Figure 3C,D). Interestingly, the concentrations of IL-4, IL-6, and IL-17A were lower in the Lp–FOS group than in the Lp and FOS groups, and there were no significant differences (Figure 3C–E).

### 3.4. Immune Cells

#### 3.4.1. DCs

**Figure 4:**
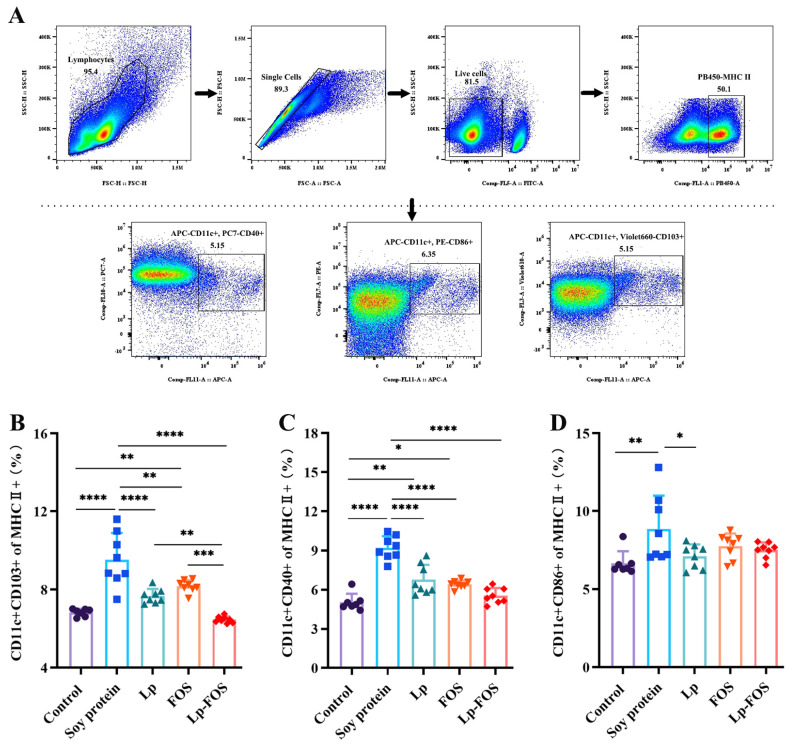
Flow cytometric analysis for DCs in the spleen. (**A**) Gating strategies of DC subsets in splenocytes. (**B**) Percentages of CD11c+CD103+ DCs. (**C**) Percentages of CD11c+CD40+ DCs. (**D**) Percentages of CD11c+CD86+ DCs. * *p* < 0.05, ** *p* < 0.01, *** *p* < 0.001, and **** *p* < 0.0001.

The activation level of DCs in freshly isolated mouse splenocytes was determined by flow cytometry (Figure 4). The results demonstrated that CD11c+CD103+, CD11c+CD40+, and CD11c+CD86+ expression levels were significantly elevated in mice in the soy protein group compared with the control group (*p* < 0.01). In contrast, CD11c+CD103+ and CD11c+CD40+ expression levels were notably reduced in the Lp, FOS, and Lp–FOS groups relative to the soy protein group (Figure 4B,C). Regarding the expression of CD11c+CD86+, a significant decrease was found only in the Lp group (*p* < 0.05, Figure 4D). Notably, compared with the Lp and FOS groups, CD11c+CD103+ expression levels were markedly decreased in the Lp–FOS group. Meanwhile, no significant differences were found in the control and Lp–FOS groups (Figure 4B). These results demonstrated that Lp and FOS were able to exert a synergistic effect and had the potential to inhibit the activation of DCs.

#### 3.4.2. Th1/Th2

**Figure 5:**
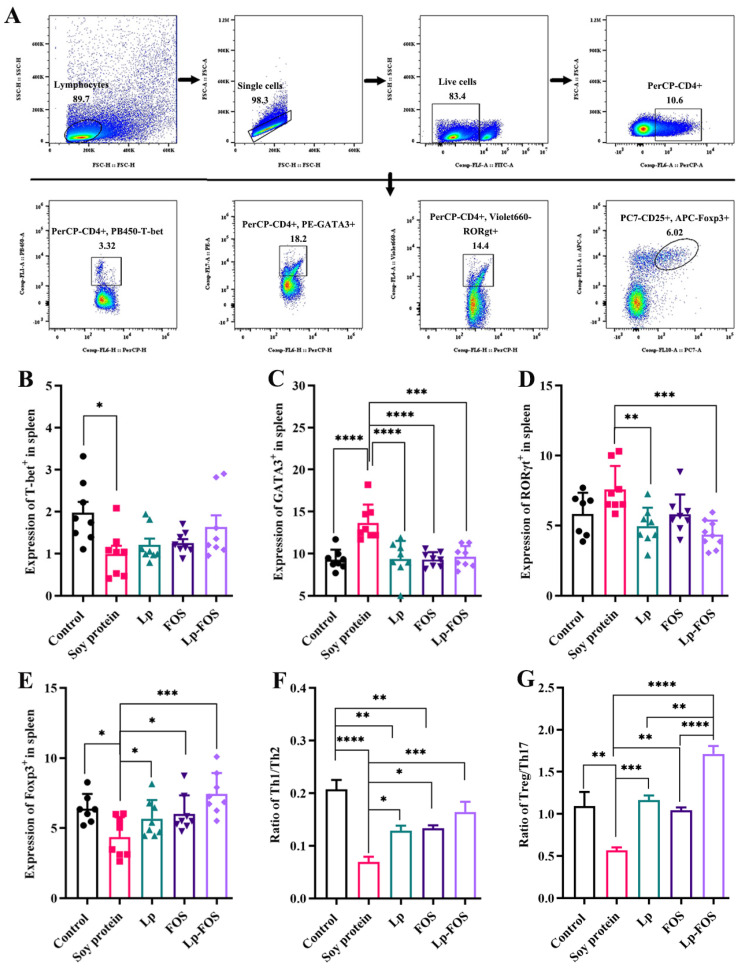
Flow cytometric analysis for T cell subsets in spleens. (**A**) Gating strategies of T cell subsets in splenocytes. (**B**) Percentage of T-bet+. (**C**) Percentages of GATA3+. (**D**) Percentage of RORγt+. (**E**) Percentage of Foxp3+. (**F**) Th1/Th2 ratio. (**G**) Treg/Th17 ratio. * *p* < 0.05, ** *p* < 0.01, *** *p* < 0.001, and **** *p* < 0.0001.

When the body experiences an allergic reaction, it is manifested as an excessive immune response by Th2 cells. The expression of Th1/Th2 cells in splenocytes was measured using flow cytometry (Figure 5B,C). It showed that the percentage of T-bet was markedly suppressed in splenocytes in the soy protein group, and no significant differences were found in the Lp, FOS, and Lp–FOS groups. However, the Lp–FOS group had the highest percentage of T-bet (Figure 5B). The percentage of GATA3 expression exhibited a significant increase in the soy protein group, with a clear difference from the other groups (Figure 5C, *p* < 0.001). Meanwhile, GATA3 expression in the control group and other intervention groups had no significant differences. Furthermore, Figure 5F demonstrated that the Th1/Th2 ratio was significantly attenuated in the soy protein group (*p* < 0.0001). The ratio of Th1/Th2 was more significantly elevated in the intervention groups than in the soy protein group, and significant differences were found in the Lp, FOS, and control groups. In particular, the Th1/Th2 ratio in the Lp–FOS group was similar to that in the control group. Taken together, these findings showed that Lp–FOS intervention markedly improved the imbalance of Th1/Th2 induced by soy protein.

#### 3.4.3. Th17 and Tregs

The expression of RORγt and CD25+ Foxp 3+ in the CD4+ cells of mouse splenocytes was identified using flow cytometry. For the expression of RORγt+ in CD4+ T cells (Figure 5D), the results illustrated that no significant differences were found between the control and soy protein groups (*p* > 0.05), but the percentage of RORγt was inhibited in the soy protein group. Compared to the soy protein group, the expression of RORγt+ was markedly inhibited in the Lp and Lp–FOS groups (*p* < 0.01 and *p* < 0.001, respectively). Compared with the control group, the expression of CD25+Foxp3+ was markedly inhibited in the soy protein group (*p* < 0.05, Figure 5E). Furthermore, it is essential to highlight that CD25+Foxp3+ expression was increased in the Lp, FOS, and Lp–FOS groups. Figure 5G showed that the Treg/Th17 ratio was suppressed in the soy protein group (*p* < 0.01). In contrast, the Treg/Th17 ratio was elevated in the Lp, FOS, and Lp–FOS groups (*p* < 0.001, *p* < 0.01, and *p* < 0.0001, respectively). It is worth noting that the Treg/Th17 ratio was also markedly increased in the Lp–FOS group. Thus, these findings demonstrated that the Treg/Th17 balance was disrupted in the soy protein-induced mouse model, but the combined Lp and FOS intervention reversed the imbalance of Treg/Th17 caused by food allergies.

### 3.5. RT-qPCR

**Figure 6:**
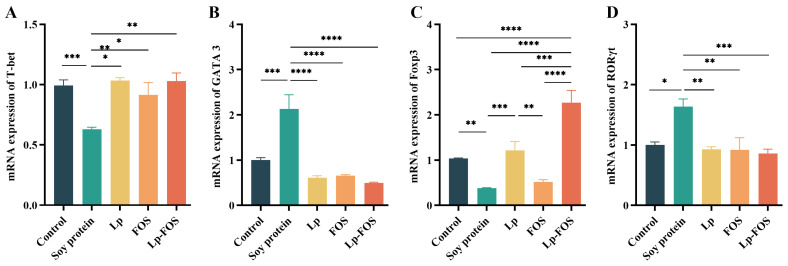
Effects of Lp–FOS intervention on the mRNA expression of transcription factors in the intestine. (**A**) T-bet. (**B**) GATA3. (**C**) Foxp3. (**D**) RORγt. * *p* < 0.05, ** *p* < 0.01, *** *p* < 0.001, and **** *p* < 0.0001. The mRNA expression levels were normalized to GAPDH housekeeping gene expression.

The effects of Lp and FOS intervention on the expression of transcription factors at the gene level were identified in the jejunum. The expression of T-bet mRNA was notably reduced in the soy protein group (*p* < 0.001). In contrast, a significant elevation in T-bet mRNA expression was detected in the Lp, FOS, and Lp–FOS groups (*p* < 0.05, Figure 6A). Furthermore, GATA3 and RORγt mRNA expression levels were also elevated in the soy protein group, while GATA3 and RORγt mRNA expression levels were markedly diminished in the Lp, FOS, and Lp–FOS groups (*p* < 0.05), and no significant differences were found compared with the control group (Figure 6B,D). In addition, Foxp3 mRNA expression was also inhibited in the soy protein group. However, an upward trend in Foxp3 mRNA expression was observed in the Lp and Lp–FOS groups. Meanwhile, the mRNA expression of Foxp3 was higher in the Lp–FOS group than in the Lp and FOS groups (*p* < 0.001, *p* < 0.0001, Figure 6C). These findings revealed that Lp–FOS expedited Th1 and Treg expression, as well as the related transcription factors T-bet and Foxp3, and suppressed Th2 and Th17 expression and the related transcription factors GATA and RORγt, which could have the potential to alleviate soybean allergic reactions and accelerate the formation of immune tolerance.

### 3.6. Lp–FOS Modulates Serum Metabolic Disorders

**Figure 7:**
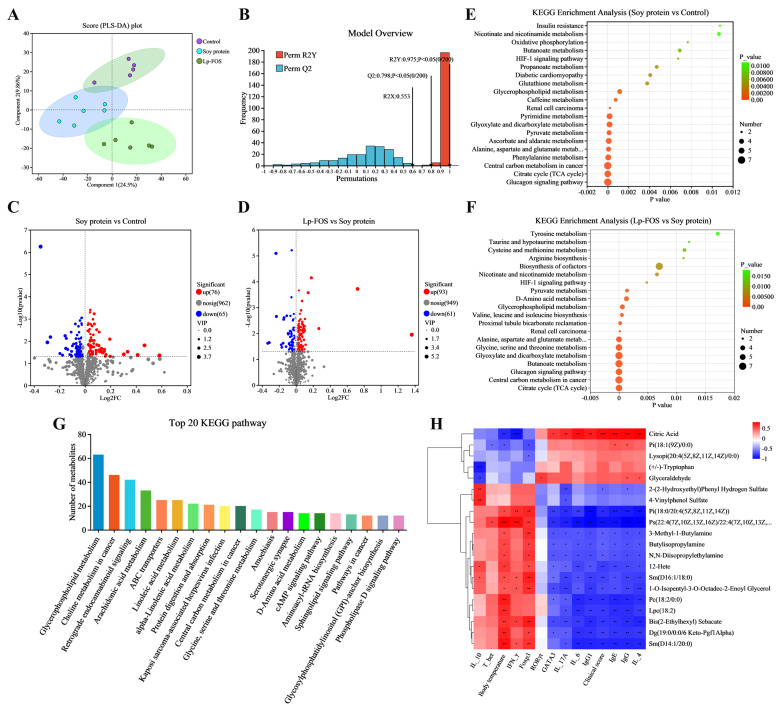
Metabolic analysis of mouse serum. (**A**) PLS-DA analysis. (**B**) Permutation tests using 200 random permutations in the PLS-DA model. (**C**,**D**) Volcano plot of soy protein vs control and Lp–FOS vs soy protein. (**E**,**F**) KEGG pathway enrichment analysis between soy protein vs control and Lp–FOS vs. soy protein. (**G**) Statistical map of the top 20 most significant pathways among all detected metabolites. (**H**) Heatmap of correlation coefficients between the potential metabolic biomarkers in serum and allergic indicators. Asterisks denote significant differences (* 0.01 < *p* ≤ 0.05, ** 0.001 < *p* ≤ 0.01, *** *p* ≤ 0.001).

Non-targeted metabolomics was performed to determine the important metabolites and metabolic pathways that were changed in the mouse serum after Lp–FOS intervention. A total of 1103 metabolites were determined in the mouse serum. PLS-DA analysis showed significant variations in principal components between the soy protein and Lp–FOS groups (Figure 7A). The R2Y (0.975) of the PLS-DA model reached up to 1.0, and the Q2 (0.798) was more than 0.5, which indicated that the model was reliable and predictable (Figure 7B). Next, differentially expressed metabolites (fold change > 1, *p*-value < 0.05, VIP-pred-OPSL-DA > 1) were further analyzed and displayed with a volcano plot (Figure 7C,D). MS/MS analysis revealed 335 different metabolites, which were determined in the serum. Specifically, compared with the control group, 141 different metabolites were determined in the soy protein group, encompassing 76 upregulated and 65 downregulated metabolites (Table S2). Furthermore, compared with the soy protein group, 154 different metabolites were determined in the Lp–FOS group, encompassing 93 upregulated and 61 downregulated metabolites (Table S3).

The identified different metabolites were sorted into metabolic sets, and pathway enrichment analysis was used to investigate potential changes in metabolic pathways (Figure 7,F). The KEGG enrichment analysis revealed that Lp–FOS intervention changed many metabolic pathways, such as glycine, serine, and threonine metabolism, butanoate metabolism, glyoxylate and dicarboxylate metabolism, the biosynthesis of cofactors, glycerophospholipid metabolism, central carbon metabolism in cancer, the glucagon signaling pathway, the citrate cycle (TCA cycle), alanine, aspartate, and glutamate metabolism, and D-Amino acid metabolism. Table 2 showed the results of KEGG pathway enrichment. Notably, the glycerophospholipid metabolism pathway was highlighted in all groups in the KEGG pathway analysis (Figure 7G).

To further study the potential relationship between serum metabolites and food allergies, the correlation between the screened differential metabolites and the allergic indicators were analyzed (Figure 7H). The findings revealed several noteworthy correlations. The levels of IgG, IL-4, clinical score, and IgE exhibited positive correlations with isocitric acid, citric acid, and 2-Furoic acid. The level of IFN-γ displayed negative associations with isocitric acid, citric acid, and 2-Furoic acid. IL-17A, IL-4, IL-6, IgG1, IgG, clinical score, and IgE exhibited a negative correlation with Pi (18:0/18:2 (9Z,12Z)) and Ps (22:4 (7Z,10Z,13Z,16Z)/22:4 (7Z,10Z,13Z,16Z). The level of IFN-γ and body temperature exhibited positive associations with Ps (22:4(7Z,10Z,13Z,16Z)/22:4 (7Z,10Z,13Z,16Z). The levels of IgG1, IgG, IL-4, clinical score, and IgE showed negative correlations with Pi (18:0/20:4 (5Z,8Z,11Z,14Z)), heptadecasphinganine, and Pc(P-18:0/18:3). The level of IL-10 was positively correlated with tyrosol glucuronide and erythritol, and negatively correlated with (+/−)-Tryptopha. These findings demonstrated that Lp–FOS might have a key role in improving particular metabolic pathways, including accelerating or inhibiting their activities. However, more comprehensive research is needed to illustrate the potential mechanisms involved.

## 4. Discussion

Synbiotics, a mixture of prebiotics and probiotics where prebiotics promote the reproduction of probiotics and colonize the gut, affecting the balance of intestinal flora and its metabolites, have been shown to alleviate food allergic symptoms [36]. It was reported that probiotics and prebiotics added to fermented milk effectively altered the gut microbiota and immunity of the host [37]. A study had shown that allergic symptoms in mice were inhibited by administrating *Bifidobacterium breve* M-16V and non-digestible oligosaccharides (scFOS and lcFOS) [38]. Therefore, the intervention effect of FOS and Lp on allergic reactions induced by soy protein was evaluated in this study. The results illustrated that FOS and Lp had a better preventive effect on soy protein-induced allergies, and it was consistent with our previous study where only *Lactobacillus* was used to intervene in soybean allergies [32].

In IgE-mediated food allergies, cytokines are important mediators for sensitization and the establishment of immune tolerance [39]. It has been shown that sensitization symptoms, specific antibodies, allergic mediators, and IL-4, IL-5, and IL-13 levels in mice were significantly elevated in the allergic state [40,41]. Studies have shown that *Lactobacillus pentosus* S-PT84 intervention attenuated allergic reactions in an egg-induced BALB/c mouse model, such as markedly alleviating the clinical allergic symptoms and attenuating histamine and mMCPT levels in the serum. Notably, *Lactobacillus pentosus* S-PT84 intervention did not affect OVA-specific IgE and IgG levels but decreased the total titers of IgE and IgG [42]. Consistent with these reports, we demonstrated that acute allergy symptoms were alleviated in BALB/c mice after administrating Lp and FOS, compared with the soy protein group, such as increased rectal temperatures, weakened allergic symptoms, and reduced serum-specific antibody levels. In addition, both the increased IL-4 level and the decreased IFN-γ level in the soy protein group verified the Th1/Th2 imbalance resulting from allergy, while Lp–FOS might ameliorate soy protein-induced allergic reactions through modulating the imbalance of Th1/Th2.

The occurrence of a food allergy involves a series of immune cells, including intestinal epithelial cells, NK cells, DCs, B lymphocytes, and T lymphocytes [43]. CD103, CD40, and CD86 were used as surface markers to detect DCs [18,44,45]. In this study, the expression of CD11c+CD103+, CD11c+CD40+, and CD11c+CD86+ were elevated in mice splenocytes after induction by soy protein, but the activation of DCs was suppressed under the intervention of FOS and Lp (Figure 4). A previous study showed that *L. murinus* enhanced IL-12 production by increasing intestinal CD11c+ and suppressing OX40 expression in OVA-induced BALB/c mice [46]. The Th1/Th2 balance has a crucial role in allergic reactions [47]. Under normal circumstances, the Th1/Th2 cells of helper T lymphocyte subsets in the body are in balance. In allergic reactions, the Th1/Th2 balance was disrupted and tilted toward a Th2 response, causing an excessive accumulation of Th2 cells in vivo [36,48]. The relevant study had confirmed that Th2 cells turned into Th1 cells and return to the equilibrium state when the allergic mice were administrated with three *Lactobacilli* [49]. *Lactobacillus helveticus* SBT2171 suppressed Th2 responses in allergic mice by inhibiting IL-4 and IL-13 levels [50]. Another study demonstrated that oral *Lactobacillus plantarum* CJLP133 and CJLP243 alleviated allergic rhinitis in mice, such as promoting a Th1-type immune response and restoring Th1/Th2 balance [51], which is consistent with our data, where Lp and FOS intervention shifted the balance of Th1/Th2 to Th1 (Figure 5B,C).

Furthermore, the occurrence of allergic reactions is related to not only the relative over-activation of Th2, but also the relative deficiency of Treg and Th17 [52,53]. Th17, as one of the T helper cells, usually has pro-inflammatory effects, while Treg is an immuno-regulatory lymphocyte and can inhibit Th2 and Th17 responses [54]. Wang et al. [55] revealed that the extracts of piper nigrum attenuated allergic reactions by inhibiting the Th2-type immune reaction and regulating the balance of Th17/Treg. In this study, it was confirmed that a diet of Lp–FOS also alleviated soy protein-induced allergies by decreasing Th17 cells, as well as increasing the percentage of CD25+Foxp3+ Tregs in the spleen (Figure 5D,E). Similar to this result, a relevant study demonstrated that FOS alleviated allergic symptoms and influenced the balance of Th17/Treg in mice by improving the composition of the gut microbiota and modulating tryptophane metabolites [14]. Furthermore, *B. lactis* intervention effectively alleviated allergic symptoms by increasing the Treg/Th17 ratio in a shrimp tropomyosin-induced mouse model [56]. In summary, the results of these studies indicated that Lp–FOS effectively alleviated the clinical symptoms of food allergies by modulating the balance of Treg/Th17, including elevating Tregs and inhibiting Th17 cells.

Additionally, the development of immune cells, including Th1, Th2, Th17, and Tregs, is determined by their corresponding transcription factors, such as T-bet, GATA-3, RORγt, and Foxp3, respectively [57,58,59]. The results revealed that Lp–FOS intervention elevated the mRNA expression levels of T-bet and Foxp3 and inhibited the mRNA expression levels of GATA3 and RORγt. A relevant study confirmed that *Lactobacillus plantarum*-CQPC11 treatment exhibited excellent effects in alleviating OVA-induced asthma, such as by upregulating T-bet and Foxp3 mRNA levels and inhibiting GATA3 mRNA levels [60]. OVA-induced airway inflammation and Th2-mediated allergic asthma were suppressed by oral oleanolic acid, including by inhibiting the transcription factors GATA-3 and RORγt and promoting the transcription factors T-bet and Foxp3 [61]. Therefore, it was reasonable to infer that Lp cooperating with FOS could suppress food allergy reactions by modulating the maturation and differentiation of immune cells.

To further validate the effect of Lp–FOS intervention, serum metabolomics analysis was performed. The clustering of different serum metabolites showed that the Lp–FOS group and soy protein group were separated, indicating that Lp–FOS intervention regulated the serum metabolism of allergic mice (Figure 7A). Compared with the soy protein group, differential metabolite analysis revealed that 154 differential metabolites were markedly affected after Lp–FOS intervention, indicating that they affected metabolite production (Figure 7D). Furthermore, KEGG pathway enrichment analysis displayed that some pathways changed in the Lp–FOS group, including glycine, serine, and threonine metabolism, glyoxylate and dicarboxylate metabolism, the citrate cycle (TCA cycle), alanine, aspartate, and glutamate metabolism, etc. (Figure 7F). Similarly, Zhao et al. [62] showed that glyoxylate and dicarboxylate metabolism, as well as glycine, serine, and threonine metabolism, was affected in allergies to honeybee venom. Xu et al. [63] demonstrated that aspartate, methionine, lysine, l-tyrosine, l-proline, and glutamate decreased in OVA-sensitized mice sera. In milk allergy and sensitized tolerance, amino acid metabolism, butanoate metabolism, and taurine and hypotaurine metabolism pathways were enriched [64]. These results revealed that Lp–FOS intervention improved the serum metabolism disorder induced by soy protein.

In summary, this study was the first attempt to establish a clinically relevant soy allergy intervention mouse model. The results of humoral immunity, cellular immunity, and serum metabolomics showed that Lp–FOS has a potential synergistic effect in alleviating soybean allergy, which may provide novel insights and solutions for soybean allergy and even food allergies in general. Secondly, BALB/c mice, as a near-crossing strain with a high IgE response, were used to establish a soy allergy intervention mouse model, which can better simulate the intervention effects of soybean allergy in clinical studies. However, there are several limitations associated with this study. (1) Based on the relationship between the gut microbiota and food allergies, it is essential to design further studies to elucidate the relationship between gut microbiota and soybean allergies. (2) Given the complexity and diversity of food allergens, it is necessary to conduct more research to explore whether Lp–FOS is the optimal combination of synbiotics for intervening in other food allergies. (3) This is basic theoretical research specifically targeting soy allergies based on animal models, and it may have some discrepancies with soybean allergies in human clinical research. Therefore, additional large-scale randomized controlled trials involving human subjects are necessary to translate these findings into clinical applications for allergy research.

## 5. Conclusions

This study clearly revealed that Lp–FOS significantly alleviated symptoms in a soy protein-sensitized mouse model. Lp–FOS intervention reduced serum-specific antibody (IgE, IgG, and IgG1) levels, promoted the release of Th1-type cytokines in splenocytes, and inhibited the secretion of Th2-type cytokines, as well as the mRNA expression of GATA-3. Meanwhile, Th1/Th2 and Treg/Th17 imbalances caused by a soy protein allergy were remediated. Furthermore, Lp–FOS intervention improved serum metabolism caused by soy protein-induced allergies. Our results demonstrated that synbiotics may be used as immuno-regulatory adjuncts in the intervention and treatment of food allergies in the future.

## Figures and Tables

**Table 1:** Clinical scores of allergy symptoms.

Score	Symptoms
0	No symptoms
1	Scratching and rubbing the nose and head less than five times;
2	Scratching the ears and nose 6–10 times, puffiness around the eyes and mouth, diarrhea, decreased activity or stationary walking, shortness of breath;
3	Scratching the ears and nose more than 10 times, wheezing, strained breathing, bruising around the mouth;
4	Convulsions, tremors, and/or seizures;
5	Shock, death.

**Table 2:** Effect of syn on KEGG pathways.

Pathway ID	Pathway Description	*p*-Value	Metabolites
map00470	D-Amino acid metabolism	0.001298	Pyruvic Acid (↓), 2-Ketoglutaric Acid (↓), L-Serine, L-Threonine (↓)
map00250	Alanine, aspartate, and glutamate metabolism	1.13^−6^	Fumaric Acid (↓), Pyruvic Acid (↓), 2-Ketoglutaric Acid (↓), Succinate Semialdehyde (↓), Citric Acid (↓)
map00564	Glycerophospholipid metabolism	0.0005883	Pc(18:2(9Z,12Z)/20:2(11Z,14Z)) (↑), Triethanolamine (↑), Ps(22:4(7Z,10Z,13Z,16Z)/22:4(7Z,10Z,13Z,16Z)) (↑), Ps(20:4(8Z,11Z,14Z,17Z)/20:0) (↑), L-Serine (↓)
map00020	Citrate cycle (TCA cycle)	2.78^−9^	Fumaric Acid (↓), L-Malic Acid (↓), Pyruvic Acid (↓), 2-Ketoglutaric Acid (↓), Citric Acid (↓), Isocitric Acid (↓)
map04922	Glucagon signaling pathway	1.61^−8^	Fumaric Acid (↓), L-Malic Acid (↓), Pyruvic Acid (↓), 2-Ketoglutaric Acid (↓), Citric Acid (↓), Isocitric Acid (↓)
map05230	Central carbon metabolism in cancer	3.95^−9^	Fumaric Acid (↓), L-Malic Acid (↓), Pyruvic Acid (↓), 2-Ketoglutaric Acid (↓), Isocitric Acid (↓), L-Serine (↓), Citric Acid (↓)
map01240	Biosynthesis of cofactors	0.007	Pyruvic Acid (↓), 2-Ketoglutaric Acid (↓), Pantothenic Acid (↓), Citric Acid (↓), Oxidized Glutathione (↓), L-Serine (↓), Isocitric Acid (↓)
map00630	Glyoxylate and dicarboxylate metabolism	2.09^−7^	Glyceric Acid (↓), L-Malic Acid, Pyruvic Acid (↓), 2-Ketoglutaric Acid (↓), Citric Acid (↓), L-Serine (↓), Isocitric Acid (↓)
map00650	Butanoate metabolism	2.30^−8^	2,3-Butanedione (↑), Butanal (↑), 3-Hydroxybutyric Acid (↑), Succinate Semialdehyde (↓), Fumaric Acid (↓), Pyruvic Acid (↓), 2-Ketoglutaric Acid (↓)
map00260	Glycine, serine, and threonine metabolism	7.67^−7^	Glyceric Acid (↓), Pyruvic Acid (↓), 2-Oxobutanoic Acid (↓), L-Threonine (↓), L-Serine (↓)

↑ and ↓ mean that the metabolites in the Lp–FOS group were higher or lower than in the soy protein group, respectively.

## Data Availability

The original contributions presented in the study are included in the article/Supplementary Material, further inquiries can be directed to the corresponding author.

## Supplementary Materials

The following supporting information can be downloaded at: https://www.mdpi.com/article/10.3390/foods14010109/s1, Table S1: qRCR-related primer sequences. Table S2: A total of 141 different metabolites between the soy protein group and the control group. Table S3: A total of 154 different metabolites between the Lp–FOS group and the soy protein group.
