# Supplementary material for: Synergistic Synbiotic-Containing Lactiplantibacillus plantarum and Fructo-Oligosaccharide Alleviate the Allergenicity of Mice Induced by Soy Protein
Source: Foods. 2025 Jan 2;14(1):109. doi: 10.3390/foods14010109 (PMC11720218; doi:10.3390/foods14010109)
Supplement: Supplementary file 1 [file foods-14-00109-s001.zip › Table S1.pdf]

Table

Table S1 qRCR related primer sequences

Gene	Forward (5'—3')	Reverse (5'—3')
GAPDH	CCTGTTGCTGTAGCCGTATTCA	CCAGGTTGTCTCCTGCGACTT
T-bet	CTGCCTACCAGAACGCAGA	AAACGGCTGGGAACAGGA
GATA3	TTATCAAGCCCAAGCGAAG	CCATTAGCGTTCCTCCTCCA
Foxp3	CCACGGGCACTATCACACAT	TTGCTTGAGGCTGCGTATGA
RORγt	ACAAATTGAAGTGATCCCTTGC	GGAGTAGGCCACATTACACTG
